# Soluble C5b‐9 (sC5b‐9) in Pediatrics—A Clinical Assessment

**DOI:** 10.1002/jcla.70122

**Published:** 2025-10-06

**Authors:** Ridwan B. Ibrahim, Radwa Almamoun, Sarah E. Sartain, Sridevi Devaraj

**Affiliations:** ^1^ Department of Pathology and Immunology Baylor College of Medicine Houston Texas USA; ^2^ Department of Pathology Texas Children's Hospital Houston Texas USA; ^3^ Department of Pediatrics, Section of Hematology Baylor College of Medicine Houston Texas USA

**Keywords:** analytical precision, complement activation, eculizumab, ravulizumab, transplant‐associated thrombotic microangiopathy, Von‐willebrand disease

## Abstract

**Background:**

The soluble C5b‐9 (sC5b‐9) is a soluble form of the Terminal Complement Complex (TCC) that is released into the circulation with elevated levels, associated with increased morbidity and mortality in patients with complement‐mediated inflammatory conditions. With the advent of eculizumab and ravulizumab, proper testing for diagnoses and therapeutic monitoring is warranted.

**Methods:**

We evaluated both the analytical and clinical performance of the Quidel Microvue sC5b‐9 Plus enzyme immunoassay. Analytical performance was evaluated with precision, linearity, interference studies, and correlation with a reference laboratory. Reference intervals were established using control donor samples [*n* = 26; median age 18.5 years (range 2–59)]. Clinical performance of the assay was assessed using plasma samples of patients who (i) developed transplant‐associated thrombotic microangiopathy [*n* = 10; median age 14 years (range 3–19)], (ii) had reduced ADAMTS13 activity [*n* = 6; median age 16 years (range 9–18)], and (iii) developed acquired Von‐Willebrand disease [*n* = 10; median age 18.5 years (range 0.5–18)].

**Results:**

The assay showed acceptable intra and inter‐precision at both low and high levels. Linearity ranged from 12.6 to 160.66 ng/mL, while accuracy and method correlation studies with a reference laboratory yielded a correlation coefficient (*R*) of 0.96. The reference range in control donors was established at ≤ 268.0 ng/mL. Clinical performance of the assay in patients' plasma revealed elevated sC5b‐9 levels suggesting complement activation in these patient cohorts compared with control levels.

**Conclusion:**

The Quidel Microvue sC5b‐9 plus EIA assay demonstrated acceptable analytical performance and clinical utility for monitoring complement activation in patients. Further studies are needed to correlate sC5b‐9 levels with existing markers of complement activation.

## Graphical abstract

The sc5b9 assay is a simple laboratory‐developed test that can be used in specific patients to monitor the course of disease, severity, or response to therapy. Future studies using this assay, which show excellent adaptability to a clinical laboratory, will reveal the multiple uses of testing for complement activation.

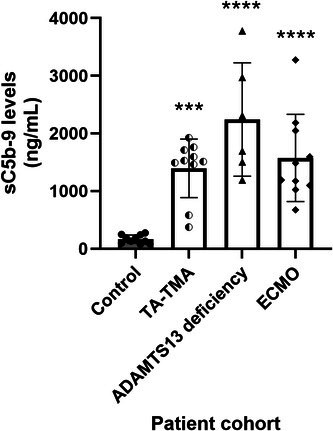

## 1 Introduction

The complement system is a crucial component of the innate immune system that contributes to cell homeostasis, tissue development, and plays an important role in protecting its host from invading pathogens [1]. The presence of pathogens leads to complement activation via three distinct pathways—the classical, lectin, and alternative pathways—all of which converge to generate the same effector molecule (C3b) through the action of the C3 convertases formed by these 3 pathways, despite having different initiating mechanisms [2]. Subsequently, these three pathways lead to the formation of the terminal C5b‐9 complement complex (TCC) [3, 4]. This cascade is tightly regulated to avoid over‐activation, which is implicated in diseases [1], such as atypical hemolytic‐uremic syndrome (aHUS), transplant‐associated thrombotic microangiopathy (TA‐TMA), etc. [5, 6]. Recently, new targeted immunotherapies such as eculizumab, ravulizumab, and zilucoplan, etc. have emerged as treatments for these conditions and have been shown to be more beneficial than traditional immunosuppressants [7, 8, 9].

Eculizumab, a complement C5 inhibitor, specifically binds to complement protein C5, inhibiting its cleavage into C5a and C5b, thereby preventing the formation of the terminal complement cascade (TCC) [10]. Laboratory monitoring of patients on eculizumab and the new C5 inhibitor ravulizumab is beneficial to assess efficacy.

Although a recent analysis of 174 acute aHUS patients reported in 14 studies showed increased sC5b‐9 levels in 93.4% of patients [11], other studies show such increased levels in only about 53% of aHUS patients [12]. Discrepant results in these studies may be due to the highly labile nature of sC5b‐9, which is important to consider when relying on plasma sC5b‐9 levels for disease monitoring or treatment, as plasma samples must be frozen quickly after blood collection and processing [13]. Used alone, it may have a high (89%) positive predictive value for active disease, but a very low (46%) negative predictive rate [14].

Currently in the United States, there are no FDA‐approved/cleared assays for sC5b‐9. One of the available assays used by clinical laboratories to measure sC5b‐9 is the Quidel Microvue sC5b‐9 EIA assay kit, which is marked for research use only (RUO) in the United States [15, 16]. In this study, we assessed the analytical and clinical performance of the Quidel Microvue sC5b‐9 plus EIA assay in our large pediatric center.

## 2 Materials and Methods

### 2.1 Quidel Microvue Test

Quidel Microvue sC5b‐9 plus EIA kits (QuidelOrtho, San Diego, CA, USA) were used throughout. The kit included manufacturer‐provided control materials (low and high) alongside five standards spanning 0–204 ng/mL. All tests were performed following the manufacturer's protocol and read using the Chromate ELISA semi‐automated analyzer (Awareness Technology Inc., Palm City, FL, USA).

### 2.2 Analytical Validation

Precision (intra‐ and inter‐assay), method comparison, analytical interferences, reference interval, linearity, and dilution studies were all assessed.

#### 2.2.1 Precision

Intra‐ and inter‐assay precision were assessed in accordance with the Clinical Laboratory Standards Institute (CLSI EP5‐A3) guidelines by measuring ten replicates of two concentration levels of the manufacturer's quality control material (mean level 1 = 27 ng/mL; mean level 2 = 153 ng/mL). Intra‐run precision was measured within a single run, while inter‐run precision was assessed once a day for 10 days. Mean concentration and coefficient of variation (%CV) were calculated for each assay.

#### 2.2.2 Linearity and Dilution Studies

The linearity of the assay was assessed by diluting the highest assay standard (assigned concentration 204) 2×, 4×, 8×, and 16× and comparing the assigned value to the observed value. Results were considered acceptable if the difference between assigned and observed was < 20%.

#### 2.2.3 Method Correlation

Accuracy and method correlation was performed by comparing sC5b‐9 assay results with 12 samples performed at a reference laboratory (Cincinnati Children's Hospital Medical Center) that uses the same sC5b‐9 ELISA kit.

#### 2.2.4 Analytical Interferences

Aliquots of pooled plasma samples were spiked with hemolysate (500 mg/dL), triglyceride‐rich lipoproteins (1000 mg/L), and conjugated bilirubin (40 mg/dL) to assess the manufacturer's claim of no interference from hemolysis up to 500 mg/dL, triglycerides up to 3000 mg/dL, and bilirubin up to 40 mg/dL.

#### 2.2.5 Reference Interval

Reference intervals for normal sC5b‐9 levels were assessed by using plasma samples from donors (*n* = 26) who are not at risk of infection, autoimmune complement activation or active inflammation.

### 2.3 Clinical Validation

Clinical performance of the assay was assessed using citrate/EDTA‐anticoagulated plasma of (i) Patients with or without a diagnosis of transplant‐associated thrombotic microangiopathy (TA‐TMA). These samples were obtained from pediatric patients undergoing bone marrow transplant (BMT) as part of two prospective cohort studies evaluating biomarkers of TA‐TMA at Texas Children's Hospital (TCH) [17, 18]. Both studies were approved by the Institutional Review Board at Baylor College of Medicine. (ii) Patients with reduced ADAMTS13 activity and (iii) Patients who developed acquired Von‐Willebrand disease from extracorporeal membrane oxygenation (ECMO).

Samples from donors and patients were obtained and processed as follows: peripheral blood was collected and anticoagulated in citrate or EDTA. Samples were centrifuged quickly (within 15 min of collection to prevent spontaneous complement activation) at 1000 × *g* for 10 min at room temperature. Plasma supernatant was aliquoted and stored at −80°C until assay. Aliquots were discarded after a single freeze–thaw (to avoid in vitro complement activation) [17].

### 2.4 Statistical Analysis

Statistical parameters (mean, SD, %CV), Deming regression, and Bland–Altman plots were generated using EP Evaluator 12.0 software. Plots were generated using Graphpad Prism 9 software.

## 3 Results

The Quidel Microvue sC5b‐9 plus EIA test demonstrated acceptable intra‐ and inter‐assay precision for both low and high controls. The %CV for the intra‐assay precision was 10.2% and 4.9% for both low and high controls, respectively (Table 1), while the %CV for the inter‐assay precision was 14.7% and 11.7% (Table 1).

**TABLE 1:** Intra and inter‐assay precision of the Quidel sC5B‐9 EIA assay using manufacturer‐assigned low and high controls.

QC material	sC5B‐9 range	Intra‐assay			Inter‐assay		
		Mean	SD	%CV (ng/mL)	Mean	SD	%CV (ng/mL)
Low control	17–37	24	2.5	10.2	28	4.1	14.7
High control	124–182	150	7.3	4.9	147	17.2	11.7

**FIGURE 1:**
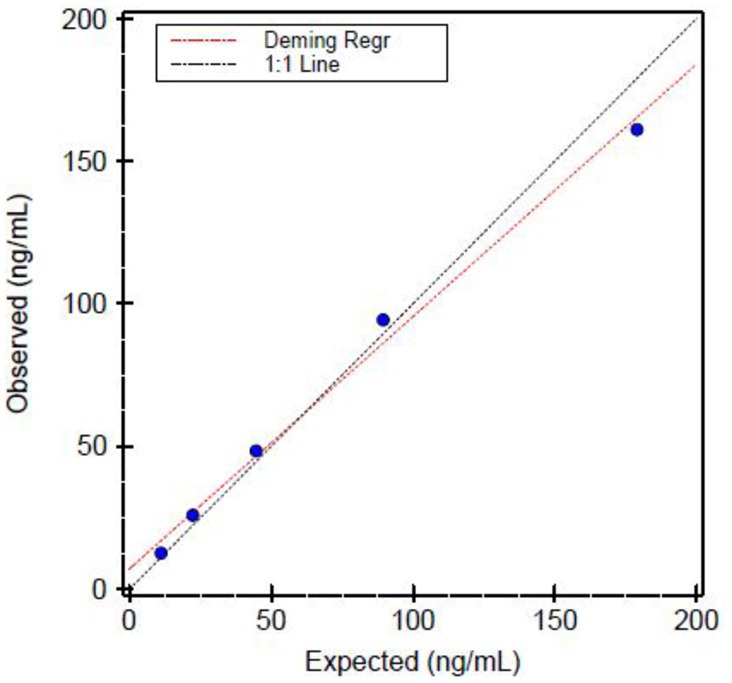
Linearity of sC5b‐9 analytical reportable range.

We analyzed the linearity of the assay (Figure 1) by preparing and testing several dilutions of high standard. The assay did not deviate significantly from linearity in the entire range of tested values. Linearity was excellent (*R* ^2^ = 0.99; intercept 7.12) in the measurement range. With a 1:16 dilution, the clinical reportable range (CRR) was assessed to extend to 2560 ng/mL.

**FIGURE 2:**
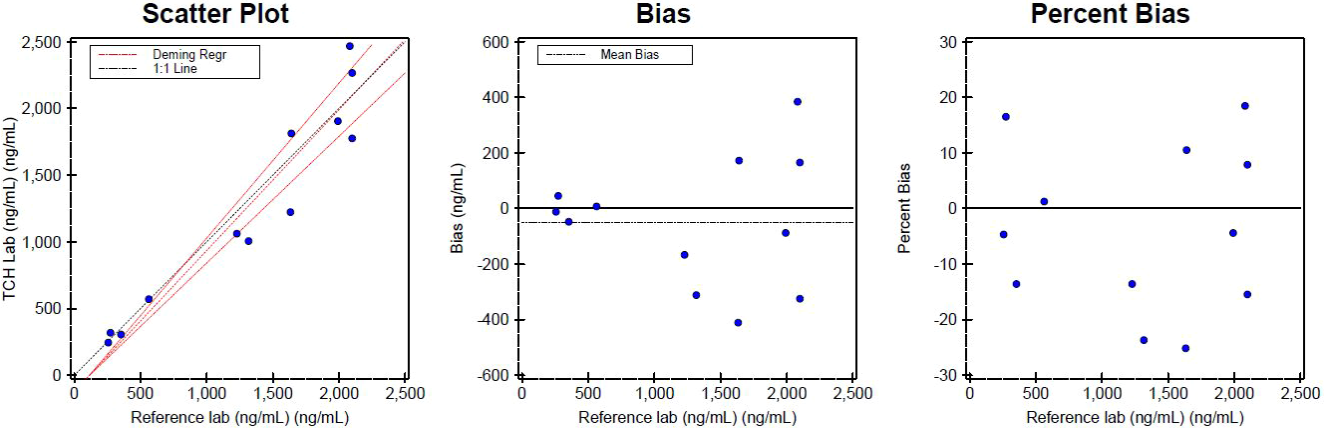
Quidel's sC5b‐9 assay comparison with a reference laboratory revealing the scatter, bias, and percent bias plots.

The accuracy and method comparison studies performed with an outside laboratory showed acceptable agreement between the two laboratories. Deming regression yielded a slope of 1.056 and an intercept of −121 with a Pearson's correlation coefficient of 0.96 and a bias of −3.8% between the two laboratories (Figure 2).

Interference studies were performed to assess the effect of common interfering substances on the sC5b‐9 assay. No significant interference was observed due to hemolysis, icterus, and lipemia at 500 mg/dL, 40 mg/dL, and 1000 mg/L, respectively (Table 2). Using a control donor specimen, we were able to verify a reference interval of < 268 ng/mL with values ranging from 79.7 to 268 ng/mL. This range was used for further clinical assessment.

**TABLE 2:** Effect of hemolysis, icterus and lipemia on Quidel sC5b‐9 EIA assay.

Interferant	sC5B‐9 level (ng/mL)	Bias	% Bias
Neat	129.2		
Hemolysis (500 mg/dL)	134.7	5.5	4.3
Icterus (40 mg/dL)	123.7	−5.5	4.3
Lipemia (1000 mg/dL)	132.6	3.4	2.6

**FIGURE 3:**
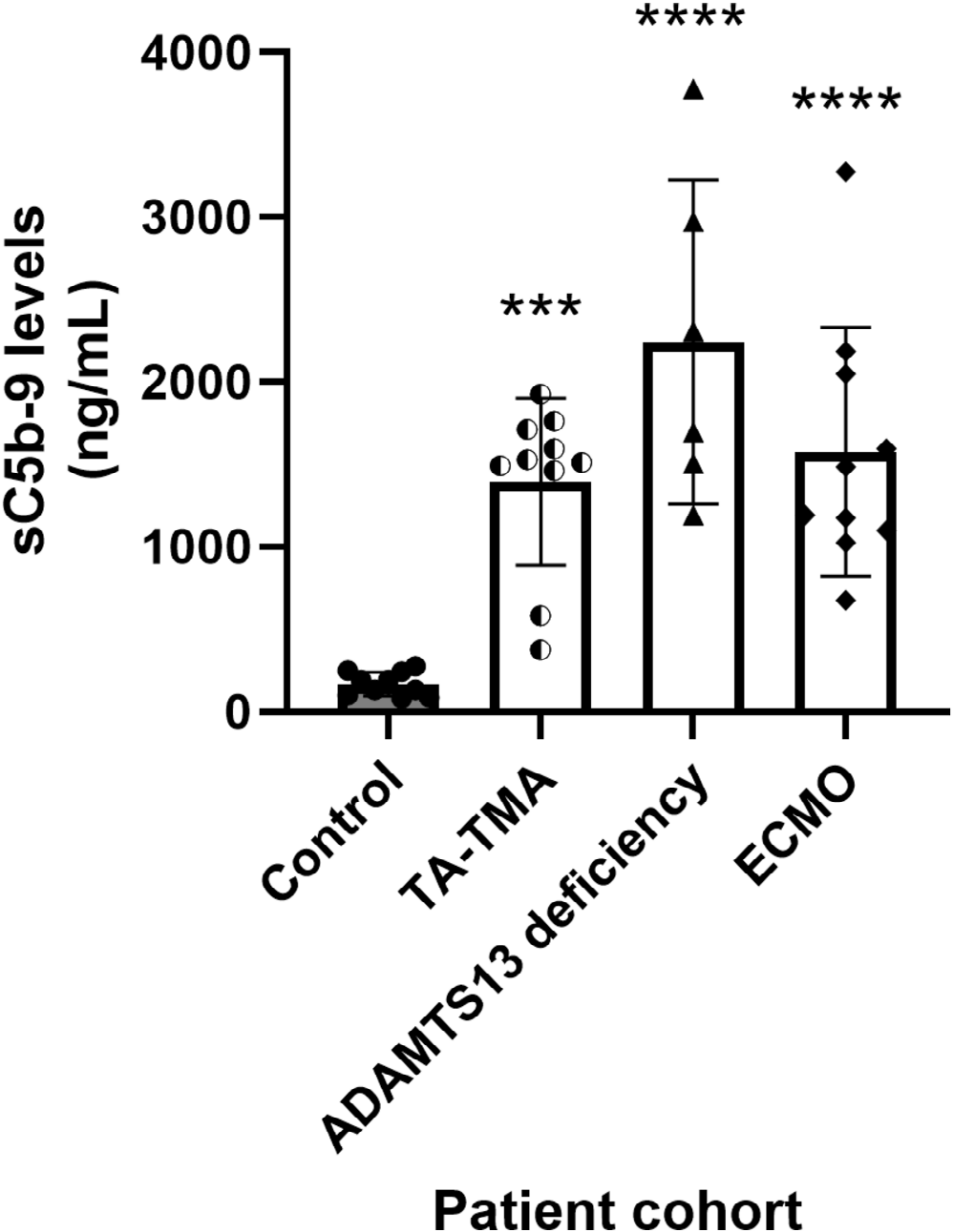
sC5b‐9 levels in control and patients who developed TA‐TMA, patients with ADAMTS 13 deficiency, and patients who developed acquired Von‐Willebrand syndrome following ECMO.

Clinical performance of the assay using EDTA‐anticoagulated plasma from (i) Bone marrow transplant (BMT) patients who developed transplant‐associated thrombotic microangiopathy (TA‐TMA), (ii) Patients with reduced ADAMTS13 activity, and (iii) Patients who were on extracorporeal membrane oxygenation (ECMO) who developed acquired von‐Willebrand disease revealed significantly elevated sC5b‐9 levels of 1394.3 ± 505 ng/mL (*p* = 0.0003), 2241.5 ± 983 ng/mL (*p* < 0.0001), and 1575.5 ± 756 ng/mL (*p* < 0.0001), respectively, suggesting complement activation in these patient cohorts compared with controls who had sC5b‐9 levels of 196.3 ± 37.4 ng/mL (Figure 3).

## 4 Discussion

In this study, we assessed the performance of the Quidel Microvue sC5b‐9 plus EIA assay in our pediatric center. Complement activation products detected in circulation can be utilized to evaluate the degree of complement activation in vivo [4, 19]. The soluble TCC (sC5b‐9), which can be detected in plasma, is the most frequently used in clinical settings [4].

Since there are no FDA‐approved/cleared assays for sC5b‐9 in the United States, we assessed the analytical and clinical performance of the Quidel Microvue sC5b‐9 EIA (QuidelOrtho, San Diego CA, USA), which is one of the available assays used by clinical laboratories to measure sC5b‐9 in a large pediatric hospital.

Our study revealed an acceptable intra‐ and inter‐assay precision range of 4.9%–10.2% and 11.7%–14.7%, respectively, which was different from the manufacturer's range of 1.9%–6.8% and 5.2%–13.1%, respectively [15]. The assay was not affected by the most commonly encountered interferences in pediatric specimens at the concentration tested (Table 2). The linearity of the assay spanned from 12.6 to 160 mg/mL, which can be extended to 2560 ng/mL with dilution, fitting for clinical applications.

Although our study shows that the sC5b‐9 assay was not affected by common pediatric interfering substances, studies have shown that pre‐analytical variables (sample processing and freeze–thaw cycles) affect complement levels [13]. A new study reveals stable sC5b‐9 levels in urine collected in the presence of protease inhibitors [20]. In contrast to an overlap in levels of plasma sC5b‐9 between active aHUS patients and controls, urine sC5b‐9 is “completely undetectable” in the urine of healthy controls [20].

We assessed the clinical utility of the assay in pediatric patients who (i) developed transplant‐associated thrombotic microangiopathy (TA‐TMA), (ii) have low ADAMTS13 activity, and (iii) develop acquired von Willebrand disease due to extracorporeal membrane oxygenation (ECMO) support. Our data reveal significantly increased levels of sC5b‐9 in all these 3 sets of patients compared to the control cohort (Figure 3).

ECMO initiation is known to be associated with inflammatory cascade activation which if unchecked may lead to endothelial injury with resultant sC5b9 elevation, and end‐organ dysfunction [21, 22]. Additionally, serum from ADAMTS13 deficient patients has been shown to induce C3 deposition and TCC formation on microvascular endothelial cells [23, 24]. Hyperactive complement activation is at the core of transplant‐associated thrombotic microangiopathy (TA‐TMA) a major complication of hematopoietic stem cell transplant (HSCT) with high morbidity and mortality [25, 26].

With the advent of the long‐acting C5 inhibitor ravulizumab for patient management [27, 28], one important use of sC5b‐9 levels might be for the assessment of effective drug levels of ravulizumab post administration. In contrast to eculizumab, which can be assessed by CH50, ravulizumab interferes with the CH50 test in vitro [27, 29], making measuring C5b‐9 levels useful. The Quidel Microvue sC5b‐9 plus EIA assay demonstrated acceptable analytical and clinical utility and can thus be utilized to monitor complement activation as part of diagnosis or in patients on therapy.

Despite the promising findings, our study is limited by small sample sizes in both the patient and control groups, which restricts the statistical power and generalizability of the findings. Further validation with larger, more diverse cohorts is necessary to confirm assay performance and better assess its clinical applicability.

In conclusion, the sc5b9 assay is a simple laboratory‐developed test that can be used in specific patients to monitor the course of the disease or response to therapy.

## Conflicts of Interest

The authors declare no conflicts of interest.

## Data Availability

The data that support the findings of this study are available from the corresponding author upon reasonable request.
